# User-Centered Design and Evaluation of Clinical Decision Support to Improve Early Peanut Introduction: Formative Study

**DOI:** 10.2196/47574

**Published:** 2023-08-22

**Authors:** Thinh Hoang Nguyen, Priscila Pereira Cunha, Annabelle Friedman Rowland, Evan Orenstein, Tricia Lee, Swaminathan Kandaswamy

**Affiliations:** 1 Department of Pediatrics Emory University School of Medicine Atlanta, GA United States; 2 Division of Immunology Boston Children's Hospital Boston, MA United States; 3 Division of Hospital Medicine Children's Healthcare of Atlanta Atlanta, GA United States; 4 Department of Allergy and Immunology Children's Healthcare of Atlanta Atlanta, GA United States

**Keywords:** clinical decision support, peanut, peanut introduction, early peanut introduction, allergy, electronic health records, simulation, user-centered design

## Abstract

**Background:**

Peanut allergy has recently become more prevalent. Peanut introduction recommendations have evolved from suggesting peanut avoidance until the age of 3 years to more recent guidelines encouraging early peanut introduction after the Learning Early about Peanut Allergy (LEAP) study in 2015. Guideline adherence is poor, leading to missed care opportunities.

**Objective:**

In this study, we aimed to develop a user-centered clinical decision support (CDS) tool to improve implementation of the most recent early peanut introduction guidelines in the primary care clinic setting.

**Methods:**

We edited the note template of the well-child check (WCC) visits at ages 4 and 6 months with CDS prompts and point-of-care education. Formative and summative usability testing were completed with pediatric residents in a simulated electronic health record (EHR). We estimated task completion rates and perceived usefulness of the CDS in summative testing, comparing a test EHR with and without the CDS.

**Results:**

Formative usability testing with the residents provided qualitative data that led to improvements in the build for both the 4-month and 6-month WCC note templates. During summative usability testing, the CDS tool significantly improved discussion of early peanut introduction at the 4-month WCC visit compared to scenarios without the CDS tool (9/15, 60% with CDS and 0/15, 0% without CDS). All providers except one at the 4-month WCC scenario gave at least an adequate score for the ease of use of the CDS tool for the history of present illness and assessment and plan sections. During the summative usability testing with the 6-month WCC new build note template, providers more commonly provided comprehensive care once obtaining a patient history concerning for an immunoglobulin E–mediated peanut reaction by placing a referral to allergy/immunology (*P*=.48), prescribing an epinephrine auto-injector (*P*=.07), instructing on how to avoid peanut products (*P*<.001), and providing an emergency treatment plan (*P*=.003) with CDS guidance. All providers gave at least an adequate score for ease of use of the CDS tool in the after-visit summary.

**Conclusions:**

User-centered CDS improved application of early peanut introduction recommendations and comprehensive care for patients who have symptoms concerning for peanut allergy in a simulation.

## Introduction

Peanut allergy is by some reports the most common food allergy among children [1,2], with most recent data showing between 2% to 5% prevalence [3,4]. The prevalence of peanut allergy has nearly doubled in the past decades, posing a growing public health concern [5-7]. Unlike many other types of food allergies, peanut allergy is rarely outgrown [8,9], leading to lifelong impact. Up to 60% of children with peanut allergy experience a severe allergic reaction [3,10-12]. Among all emergency department (ED) visits and hospitalizations for anaphylaxis, including in intensive care units, peanut products are the most common trigger. Although fatality is not common, morbidity and reductions in quality of life remain a concern [13]. In one study, children with peanut allergy reported poorer quality of life than children with insulin-dependent diabetes [14].

In 2000, the American Academy of Pediatrics released guidance suggesting avoidance of peanuts until the age of 3 years [15], but later revised this, stating in 2008 there was inconclusive data regarding the benefits of delaying peanut introduction [16]. In 2015, the Learning Early about Peanut Allergy (LEAP) study showed that early introduction of peanuts between the ages of 4 months and 11 months was not only safe but also led to lower rates of peanut allergy among high-risk infants [17]. Another study, the Enquiring About Tolerance (EAT) trial, expanded on the previous results by showing that early peanut introduction in infants from a general population, regardless of risk, led to a lower prevalence of peanut allergy [18]. Based on these results, the National Institute of Allergy and Infectious Diseases (NIAID) released new guidelines in 2017, recommending early introduction of peanuts according to risk stratification [19].

Despite the new guidelines, the rates of early peanut introduction remain low [20-23]. The recommendation for testing high-risk infants prior to early introduction created confusion [21,24], high cost [25], and logistical challenges [21,24,25]. Other barriers to implementation among pediatricians included lack of awareness of guidelines and low comfort discussing them with parents [21,24]. Caregivers have reported fear of introducing peanut products or unawareness of the data, with many saying they relied on their pediatrician for such recommendations [23,26-28]. Interestingly, in Australia, where guidelines recommend introduction around the age of 6 months for all individuals and do not include any testing, there was a 3-fold increase in peanut product introduction before the age of 1 year [29]. In order to address some of the concerns, in 2021 consensus guidance was released by 3 major North American allergy organizations recommending introduction of peanuts to all infants around 6 months of age, with less emphasis on allergy testing [30].

Though most infants will not have symptoms from peanuts, some may have symptoms suggestive of an immunoglobulin E (IgE)–mediated allergy, which can cause severe, life-threatening anaphylactic reactions. The American Academy of Allergy, Asthma, and Immunology (AAAAI) outlines anticipatory guidance that clinicians should provide to patients and families who are at risk of anaphylaxis. The guidelines include educating patients about anaphylaxis, the risk of recurrence, trigger avoidance, and thresholds for future care, as well as providing a prescription for an epinephrine auto-injector (EAI) and a referral to an allergist [31].

Although there is not much data available pertaining to the prescription rates of EAIs for patients with a first-time reaction specific to peanuts, one meta-analysis looked at EAI prescription rates for patients with anaphylaxis regardless of the allergic trigger. [32]. Of the 75 studies reviewed, 44 reported less than 50% of the patients who had experienced anaphylaxis had a prescription for an EAI [32]. It remains crucial that EAIs are prescribed at time of diagnosis of any IgE-mediated allergy. More information is needed regarding the prescription patterns of EAIs by pediatric primary care providers for peanut-allergic patients.

Despite these challenges, one study, from Clark et al [33], described an improvement in providing anticipatory guidance in the ED for patients presenting with food-induced anaphylaxis over a 15-year interval period. Overall, physicians prescribed EAIs, gave allergy referrals, and provided allergen avoidance education at statistically significant higher rates when compared to 15 years prior. However, only 23% of patient encounters met criteria for at least 3 or more of the AAAAI guidelines [33]. This study emphasized the need for addressing provider variability despite standardized guidelines and closing gaps between recognizing food-induced anaphylaxis and taking the steps to prevent its recurrence with proper anticipatory guidance. Little information is available about the success rates of providers in the primary care setting in adhering to the main AAAAI anticipatory guidelines for IgE-mediated reactions, especially in the setting of peanut allergy.

Clinical decision support (CDS) provides timely point-of-care information to clinicians, who can then share decision-making with patients. CDS design often suffers from poor usability and consideration of workflows [34,35], leading to poor adoption and ultimately to inconsistent effectiveness of CDS [36-38]. Adopting human factors principles and following a user-centered design process with iterative design based on feedback from users and usability evaluations can improve effectiveness of CDS by improving usability as well as ensuring appropriate workflow integration [39-41].

The aims of this study were to (1) apply user-centered design in a formative evaluation to design and develop a CDS tool to recommend early peanut introduction and (2) evaluate effectiveness of the CDS tool in simulated settings.

## Methods

### Setting

The study was conducted at a large academic pediatric primary care clinic within an inner-city freestanding children’s hospital serving diverse, low-income, and historically marginalized populations.

### Ethical Considerations

This study was a quality improvement project and was deemed non–human subject research by the institutional review board at Children’s Healthcare of Atlanta (STUDY00001218).

### CDS Development

We developed an initial prototype of the CDS tool after brainstorming with the CDS team and clinical stakeholders. This CDS tool was built as a collection of automated prompts within the well-child check (WCC) note, some of which included reminders with disappearing text that was automatically deleted at the signing of the note [42] with branching logic within the progress note template in a test electronic health record (EHR) environment.

### Formative Usability Testing

We adopted a think aloud protocol and conducted formative usability testing to iteratively redesign our CDS tool to promote early peanut introduction [43,44]. We reached out to participants in-situ, that is, in their clinical workspace, and asked if they had 10 minutes to provide feedback on a tool for decision support. Participants were free to attend clinical calls or step out for patient care if needed in between the testing sessions. We briefly explained that the goal of the study was to obtain feedback on a CDS tool without explicitly mentioning anything about peanut introduction. After providing them with psychological safety, we provided them with a clinical scenario of an otherwise well child who breastfeeds. Participants were asked to think aloud as they completed documentation of the diet section on a mock patient chart. They were free to ask questions to one of the study team members administering the test who acted as the parent. At the end of documentation participants were debriefed on the aim of the study and qualitative feedback on design of the CDS tool was obtained, with notes checked by a team member with the participant. Based on feedback, we iteratively improved design between participants before summative usability testing.

### Summative Usability Testing

To estimate the effectiveness of the CDS tool, we conducted summative testing. An updated CDS design based on learnings from the formative testing was used. Similar to formative testing, we reached out to participants in-situ, that is, in their clinical workspace, and asked if they had 10 minutes to test a tool for decision support. Participants were given 1 of the 2 clinical scenarios and were asked to complete the documentation as they would in practice, first with the existing design and then with the updated design for the same scenario. At the end of the test, participants completed a survey with a 4-point Likert scale (1=“not useful,” 2=“adequate,” 3=“useful,” 4=“very useful”) on the usefulness of the CDS tool. They were also asked to provide qualitative feedback. One test administrator took notes and at the end of the testing notes from the feedback were checked by a team member with the participants. One of the test administrators acted as a parent and answered participant questions. Scenario 1 was a 4-month WCC visit with focus on the diet that included breastfeeding. For scenario 1, we defined a task as successfully completed if the participant talked about peanut introduction and discussed options for peanut introduction. Scenario 2 was a 6-month WCC visit that included the parent providing new history that the infant tried peanuts and had symptoms suggesting an IgE-mediated reaction. For scenario 2, we evaluated successful task completion based on 4 actions, including referring to allergy/immunology, prescribing an EAI, instructing on how to avoid peanuts, and providing an emergency treatment plan.

### Statistical Analysis

All statistical analyses were performed using R (version 3.6.3; R Core Team). We used descriptive statistics to summarize task completion rates with and without CDS in the summative testing. Significant differences in performance were assessed at a *P* value of .05 using the McNemar test.

## Results

### Formative Usability Testing

**Figure 1:**
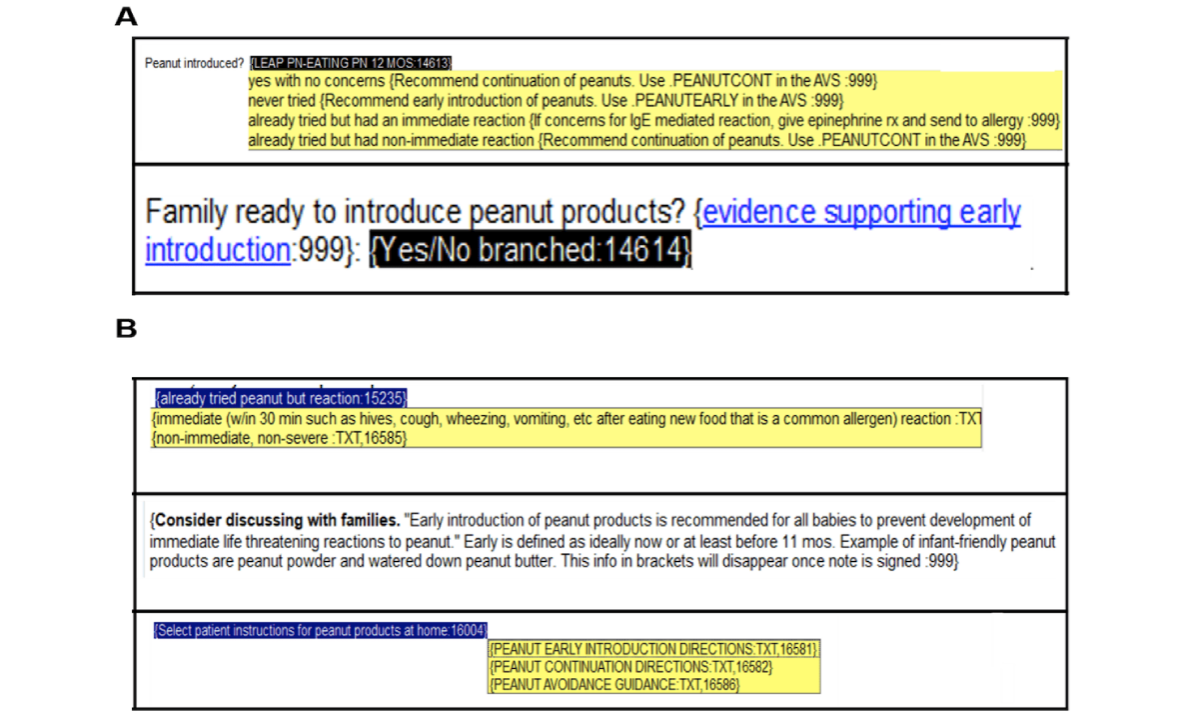
Screenshots of (A) the original design of the clinical decision support tool and (B) the edited design after major changes were made based on formative testing. Feedback included comments that the tool needed to include point-of-care education on the definition of an immediate reaction, that the hyperlink was confusing and tedious, and that it was possible to misunderstand the instructions for the after-visit summary.

A total of 6 pediatric residents participated in the formative testing (2 interns, 3 third-year residents, and 1 chief resident). All 6 residents reported that the CDS tool was overall useful and easy to use. The main concerns from residents were that the tool may need to provide point-of-care education on the definition of an immediate reaction, that the inclusion of a hyperlink that took the user to the literature supporting early peanut recommendation was confusing and tedious, that it was possible to misunderstand the instructions on how to include the early peanut introduction guidance in the after-visit summary (AVS), and that they desired documentation assistance in the plan section of the note about the early peanut introduction discussion with the family. Based on this feedback, 4 major build changes were made to the CDS tool: (1) a disappearing text description with the definition of an immediate reaction was added; (2) the hyperlink to a specific citation was replaced with disappearing text showing a 1-sentence summary of the recommendations; (3) a description of the discussion with the family regarding peanut introduction was added to the plan section; and (4) a drop-down menu was added that automatically populated in the AVS and allowed the user to select instructions for either peanut introduction, continuation, or avoidance (Table 1 and Figure 1).

**Table 1:** Major changes to the clinical decision support tool after formative testing.

Original design	Resident feedback	Edited design
At the 6- and 12-month visits, when discussing peanut introduction, if the family had already tried peanut products and reported a reaction, residents were supposed to document if it was an immediate or nonimmediate reaction.	Residents reported that they may need point-of-care education on the definition of an immediate reaction.	A disappearing text description was added with the definition of an immediate reaction.
When assessing family readiness and planning introduction of peanut products, residents were given a hyperlink that took them to literature supporting early peanut introduction.	Residents found the inclusion of a hyperlink was confusing and tedious.	The hyperlink was replaced with a disappearing 1-sentence summary of the recommendations.
Residents freely typed what they discussed with the family in the note’s assessment and plan section.	Residents desired documentation assistance in the plan section of the note about the early peanut introduction discussion with the family.	A description of the discussion with the family regarding peanut introduction was added to the plan section.
At 4-, 6-, and 12-month visits, residents were given a SmartPhrase (an abbreviation that helps to pull long phrases into a note in the Epic system) to type into the after-visit summary that would insert peanut introduction, continuation, or avoidance instructions.	Residents misunderstood the instructions on how to include the early peanut introduction guidance in the after-visit summary.	A drop-down menu to select instructions for either peanut introduction, continuation, or avoidance automatically populated the after-visit summary.

### Summative Usability Testing

A total of 30 providers (Table 2) participated in the summative testing—15 for scenario 1 and 15 for scenario 2 (Table 3 and Table 4). The demographics of participants in our summative testing are provided in Table 2. The task completion rate in scenario 1 was significantly higher with CDS, at 60% (9/15), compared to 0% without CDS (0/15; *P*=.008).

For scenario 2, all participants placed an allergy/immunology referral when using CDS while 2 of 15 did not refer patients with the original note template that did not contain the CDS (*P*=.48). A total of 87% (13/15) prescribed an EAI when using CDS, while only 53% (8/15) did without CDS (*P*=.07). There was significant difference in users providing instructions on peanut avoidance, where 100% (15/15) of participants provided anticipatory guidance when using CDS while only 13% (2/15) provided such instruction without CDS (*P*<.001). All participants provided an emergency treatment plan when using CDS, while only 26% (4/15) completed this element without CDS (*P*=.003). All participants completed at least 3 of 4 elements in the AAAAI guidelines with CDS, compared to 33% (5/15) without CDS.

**Table 2:** Summative testing participant demographics (n=30).

Characteristics			Participants, n (%)
**Role**			
	Attending	4^a^ (13)	
	Resident	26 (87)	
**Sex**			
	Male	10 (33)	
	Female	20 (67)	
**Race**			
	White	19 (63)	
	Black	5 (17)	
	Asian	5 (17)	
	Hispanic/Latino	1 (3)	
**Experience (years)**			
	>10	2 (7)	
	6-7	1 (3)	
	4-5	1 (3)	
	2-3	8 (27)	
	1-2	12 (40)	
	<1	6 (20)	

^a^Including 1 nurse practitioner.

**Table 3:** Scenario 1: Participants who discussed peanut introduction with and without CDS^a^.

	Participant														
	1	2	3	4	5	6	7	8	9	10	11	12	13	14	15
Without CDS															
With CDS	✓^b^	✓						✓	✓	✓		✓	✓	✓	✓

^a^CDS: clinical decision support.

^b^✓: indicates that the participant successfully completed that specific task.

**Table 4:** Scenario 2: Actions completed by participants with and without clinical decision support. Checkmarks in the rows labeled “Successful” indicate that a participant completed at least 3 of 4 elements in the American Academy of Allergy, Asthma, and Immunology guidelines.

			Participant completed the action																												
			1		2		3		4		5		6		7		8		9		10		11		12		13		14		15
Actions																															
**Without clinical decision support**																															
	Allergy/immunology referral	✓^a^		✓		✓		✓		✓		✓				✓		✓		✓		✓		✓				✓		✓	
	Prescription for epinephrine	✓		✓		✓				✓				✓				✓						✓						✓	
	Instructions to avoid peanuts					✓												✓													
	Discuss treatment plan	✓								✓								✓												✓	
	Successful	✓				✓				✓								✓												✓	
**With clinical decision support**																															
	Allergy/immunology referral	✓		✓		✓		✓		✓		✓		✓		✓		✓		✓		✓		✓		✓		✓		✓	
	Prescription for epinephrine	✓		✓		✓				✓				✓		✓		✓		✓		✓		✓		✓		✓		✓	
	Instructions to avoid peanuts	✓		✓		✓		✓		✓		✓		✓		✓		✓		✓		✓		✓		✓		✓		✓	
	Discuss treatment plan	✓		✓		✓		✓		✓		✓		✓		✓		✓		✓		✓		✓		✓		✓		✓	
	Successful	✓		✓		✓		✓		✓		✓		✓		✓		✓		✓		✓		✓		✓		✓		✓	

^a^✓: indicates that the participant successfully completed that specific task.

### Qualitative Feedback From Summative Testing

Participants had feedback to improve the CDS design. First, they needed information on infant-friendly peanut products that they could suggest to patients’ families. Though this information was available in the AVS, they wanted the information accessible in the progress note at the point-of-care interaction with the family. Second, participants felt the term “early” in reference to the timing of peanut introduction was vague and wanted better-defined parameters. Third, one senior attending health care provider felt that the CDS prompt asking to read the sentence about early peanut introduction was burdensome and inefficient. She instead wanted a hard-stop yes-no question that providers could answer in the note. She felt that would enable her to bring up the topic as they liked but act as a reminder and a place to document conversation about early introduction of peanuts. Fourth, some participants who had missed noticing the CDS tool suggested adding some highlighting elements draw their attention to reading the sentence. Fifth, participants wanted clarifying verbiage in the progress note to refer to the AVS for the peanut introduction instructions. Sixth, participants were confused by the command- or code-like appearance of the note template and asked for clarification. Seventh, participants also suggested clarifying language in the note template to distinguish if the plan to introduce peanuts was mutually agreed between providers or if there was a disagreement between the provider and patient. Eighth, participants also wanted information, likely in the assessment/plan (A/P) section, indicating when peanuts should not be introduced. Ninth, participants suggested to move the prompt to the top of the history of present illness (HPI) section instead of diet so that providers who did not use the diet section could still see and benefit from the CDS. Tenth, participants who missed seeing the CDS tool when talking with family mentioned that CDS was helpful even if they missed it during the simulation. They said that if they saw it at least twice, they were likely to remember the need to answer this section in the note template. They cited examples of similar interventions that needed documentation in other parts of the EHR that became part of their workflow.

### Survey Response

**Figure 2:**
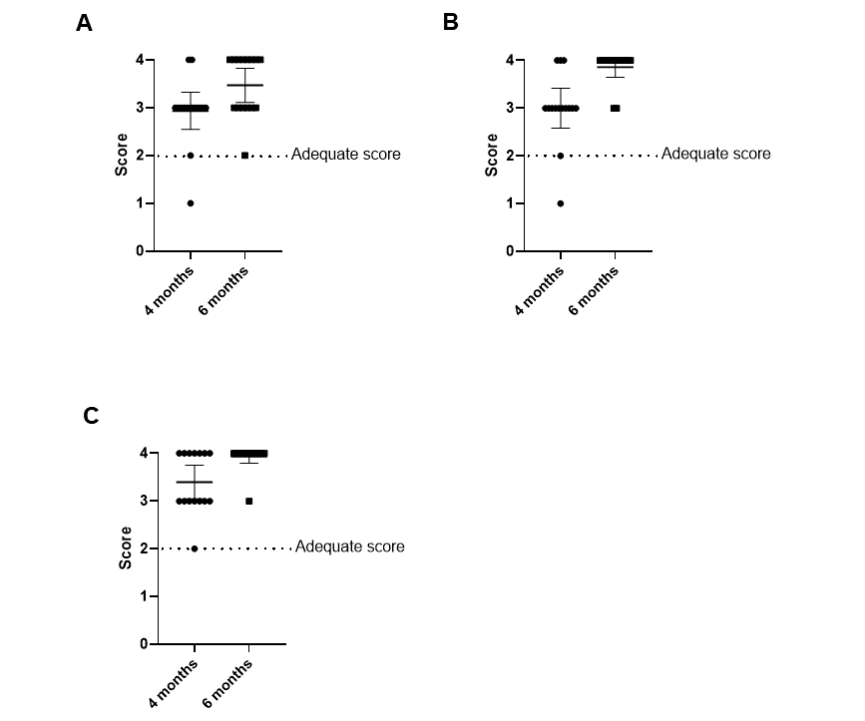
The ease of use of clinical decision support for 4-month well-child check and 6-month well-child check scenarios for (A) history of present illness, (B) assessment and plan, and (C) after-visit summary. Participants were given 4 different choices, including 1=“not useful,” 2=“adequate,” 3=“useful,” and 4=“very useful.”.

Participants felt that the CDS tool was easy to use (mean score 3.5 on a scale of 1=“not useful,” 2=“adequate,” 3=“useful,” and 4=“very useful”). The distribution of participant responses to the usefulness of the CDS tool is shown in Figure 2. For the question “Does this clinical decision support help to introduce the topic of peanut introduction to families?” 87% (13/15) of participants gave a response of “useful” or “very useful.” Similarly, 87% (13/15) of participants gave a response of “useful” or “very useful” when asked “Does this automated smartphrase in the AVS about how to introduce peanut products help you to know what is important to tell families?” In the 6-month WCC scenario, all participants indicated that the CDS was at least adequate for helping them to remember to order an allergy referral. All participants responded “useful” or “very useful” when asked about the usefulness of the CDS in reminding them to prescribe an EAI. They also indicated that the automatic SmartPhrase in the AVS was useful to remind them what was important to tell families (mean score 3.47 in the 4-month WCC scenario, mean score 3.8 in the 6-month WCC scenario).

## Discussion

### Principal Results

In this study, we applied user-centered design to develop a CDS tool for early peanut introduction and evaluated the effectiveness of the CDS tool in simulated settings. Formative testing revealed areas for improvement of the tool, such as providing more active point-of-care education to the user in the tool and adding repeated, consistent reminders throughout the note template. We observed that formative testing not only helped inform user-centered design, but also increased user investment in the tool. Key design changes from the qualitative feedback during summative testing included moving the placement of the tool in the established note template higher up to allow for better visibility, improving the uniformity of the language, and adding more point-of-care education. In the summative evaluation, the improved CDS design successfully facilitated discussions on early peanut introduction during 4-month WCC visits. Furthermore, adherence to guideline recommendations improved for the scenario in which the patient had a history of IgE-mediated peanut allergy at the 6-month WCC. This is evidenced by all participants completing at least 3 of the 4 elements in summative testing with CDS, compared to only 23% in past literature [33].

Although the majority of participants integrated the CDS tool into their workflows, complete adoption was not achieved. This was likely due to differences in provider workflows. Specifically, some providers completed the documentation at the desk away from the family after speaking with the parent, while others completed the documentation while speaking with the family. During summative testing, providers who followed the former workflow did not see the CDS tool in the simulation and therefore did not use it. While this issue may not be completely resolved, we believe that the user-friendly CDS tool, developed with user-centered design and with a note template and education module, could improve utilization rates during real-life implementation.

Despite recent rapid guidelines changes, there is still a dearth of literature on CDS development and implementation in the field of peanut allergy prevention and treatment, specifically at the primary care level [45]. While there is some data on the implementation of feeding guidelines in Australia, it is unclear whether any informatics techniques were used to encourage these practice changes. There continues to be data to support the lack of implementation of the early peanut recommendations by pediatricians specifically here in the United States [21,27,46]. However, interventions such as the Intervention to Reduce Early (Peanut) Allergy in Children (iREACH) program have been successful in improving pediatrician adherence to early peanut introduction guidelines in Chicago, Illinois [47]. This group has not published the exact build details of their CDS tool, except to say that it includes an order set, prompts, handouts, and a best-practice advisory (BPA). Generally, BPAs are interruptive to workflows. Our build development may be unique in that it has a user-centered design based on formative and summative usability testing. Also, our CDS tool is automated in all WCC encounters, is noninterruptive, provides point-of-care education with disappearing text, and uses branching logic. Moreover, our CDS tool implements more recent consensus recommendations on peanut introduction that do not stratify patients by risk.

A prior attempt to improve management of food allergy patients in one health system via an interruptive alert in the EHR (triggered by either a diagnosis code of food allergy or EAI prescription) found no significant difference in allergy referral, EAI prescription, or food allergy action planning [48]. While the most common form of CDS tool is an alert, these are not always effective to drive process change or improve outcomes [49]. Furthermore, they are interruptive in nature, adding to alert fatigue and clinician burnout [50]. Our passive CDS tool avoids the risk of alert fatigue but was able to achieve process change, as evident from simulated tests and the favorable scores in the user survey of the final build of our CDS tool. Future real-world scenario testing would further confirm the rate of adoption for our build.

### Limitations

This study has some limitations. First, the study is limited by the evaluation being performed at a single institution. Second, while we used realistic patient scenarios and had study team members act as family to answer questions, we did not go through the whole visit in simulation. This may have biased conversation about peanut introduction. However, this risk is low, as we confirmed with participants if they would have bought it up in conversation to parents at another time during such an encounter, and they responded negatively. Third, all participants in the formative testing were residents. This may have led us to miss some of the workflow considerations of attending physicians in the CDS design. However, we captured feedback from attending physicians in summative testing and will accommodate them with design changes prior to real-world implementation. Finally, we are limited by the lack of evaluations in clinical practice. As a next step, we plan to test the CDS tool in a real-world clinical setting, which should show the effectiveness of the tool in supporting early peanut introduction as well as standardizing care for newly presenting peanut reactions.

### Conclusion

Passive CDS tools, as opposed to active alerts, can be effective to help implement evidence-based practices for all patients despite the burdens of busy clinics. User-centered design with feedback from clinical users and rapid prototyping can help develop solutions that work for users and will be seamlessly incorporated into their existing workflows, thus changing clinical outcomes.

## Abbreviations

A/P: assessment and plan
AAAAI: American Academy of Allergy, Asthma, and Immunology
AVS: after-visit summary
BPA: best-practice advisory
CDS: clinical decision support
EAI: epinephrine auto-injector
EAT: Enquiring About Tolerance
ED: emergency department
EHR: electronic health record
HPI: history of present illness
IgE: immunoglobulin E
iREACH: intervention to Reduce Early (Peanut) Allergy in Children
LEAP: Learning Early about Peanut Allergy
NIAID: National Institute of Allergy and Infectious Diseases
WCC: well-child check

## Data Availability

The data sets generated during and/or analyzed during this study are available from the corresponding author on reasonable request.
